# Supervised extraction of near-complete genomes from metagenomic samples: A new service in PATRIC

**DOI:** 10.1371/journal.pone.0250092

**Published:** 2021-04-15

**Authors:** Bruce Parrello, Rory Butler, Philippe Chlenski, Gordon D. Pusch, Ross Overbeek

**Affiliations:** 1 Fellowship for Interpretation of Genomes, Burr Ridge, IL, United States of America; 2 University of Chicago, Chicago, IL, United States of America; University of Helsinki, FINLAND

## Abstract

Large amounts of metagenomically-derived data are submitted to PATRIC for analysis. In the future, we expect even more jobs submitted to PATRIC will use metagenomic data. One in-demand use case is the extraction of near-complete draft genomes from assembled contigs of metagenomic origin. The PATRIC metagenome binning service utilizes the PATRIC database to furnish a large, diverse set of reference genomes. We provide a new service for supervised extraction and annotation of high-quality, near-complete genomes from metagenomically-derived contigs. Reference genomes are assigned to putative draft genome bins based on the presence of single-copy universal marker roles in the sample, and contigs are sorted into these bins by their similarity to reference genomes in PATRIC. Each set of binned contigs represents a draft genome that will be annotated by RASTtk in PATRIC. A structured-language binning report is provided containing quality measurements and taxonomic information about the contig bins. The PATRIC metagenome binning service emphasizes extraction of high-quality genomes for downstream analysis using other PATRIC tools and services. Due to its supervised nature, the binning service is not appropriate for mining novel or extremely low-coverage genomes from metagenomic samples.

## Introduction

Improvements in sequencing and assembly technology have created a wealth of metagenomic sample data. Because most strains occurring in the world have not been successfully cultured in laboratory conditions, many species’ genomes are being reconstructed for the first time from metagenomic samples. This represents an important step towards a more complete characterization of true phylogenetic diversity and supports more accurate comparative analysis of microbial genomes.

In a recent paper, Pasolli *et al.* assembled over 153,000 high- and medium-quality draft genomes from metagenomic samples [1], demonstrating the possibility of high-throughput metagenomic genome mining and indicating a future direction of data generation in genomics. It should be expected that, going forward, most new genomes being made available will be metagenomic in origin. The recently announced “Million Microbiome of Humans Project” suggests the scale of microbiome data that will become available in the near future.

In addition to genome discovery, rapid and accurate metagenomic sample characterization is in demand for a variety of industries including agriculture, ecology, and medicine. In particular, the ability to extract reasonably complete draft genomes from hospital samples without investing in specialized computing infrastructure is an important tool for diagnosis and research.

A key computational challenge in metagenomics is the binning of assembled contigs into draft genomes. The high abundance of repeat regions, presence of DNA from low-abundance populations in the sample, inherent noisiness caused by the presence of multiple species in a single sample, and the lack of good reference genomes for unculturable populations make it challenging to produce full-length genomes from metagenomic reads [2]. Many of these problems affect metagenomic assembly, e.g. through assembly of chimeric contigs, and then propagate these problems into the subsequent binning step [3]. All of these factors contribute to the need for specialized binning tools and high computational demands. Moreover, existing tools for metagenome binning either lack a stable online implementation or lack integration into a bioinformatics database which could support downstream analysis.

PATRIC, the Pathosystems Resource Integration Center, is a web-based service providing omics data and analysis tools that support the analysis of prokaryote genomes [4]. At the time of publication, it contains 324,422 annotated bacterial genomes and 4,807 annotated archaeal genomes. This number is expected to double in the next year. All of the genomes are annotated using RASTtk [5] to provide a single, controlled vocabulary for annotation, as well as a suite of quality metrics such as EvalG and EvalCon [6] for assessing draft genome quality. This controlled vocabulary enables us to make detailed comparative quality assessments among genomes. Such assessments are a crucial tool for the interpretation and evaluation of metagenome binning results.

Metagenomic analysis is in demand as a bioinformatics service, and most state-of-the-art services have high overheads in terms of configuration and investment in specialized computing resources. We aim to provide PATRIC users with an easy-to-use, low-overhead, detailed, rapid, and structured metagenome binning service emphasizing extraction of high-quality genomes for downstream analysis in PATRIC. Although it will likely be possible to achieve better performance on any given dataset using bespoke methods and specialized tools, our goal is to achieve near-state-of-the-art performance in as many cases as possible. However, the binning service is not designed for mining novel or low-coverage genomes, nor does it take advantage of coverage information (e.g. to provide relative clade abundances). Users seeking to perform such tasks may need to consider other PATRIC tools such as the read mapping and taxonomic classification service or third-party services.

## Materials and methods

The PATRIC metagenome binning service takes metagenomic samples as assembled contigs or paired-end reads, extracts and annotates whole genomes, places them in the user’s workspace, and indexes them in the PATRIC database as private genomes. If reads are submitted, the metagenome binning service first assembles them into contigs using metaSPAdes. [7] Contigs are then binned using a novel supervised binning algorithm in which reference genomes are selected by searching for universal roles in the sample. (A genome contains a set of protein-encoding genes, or PEGs. These encode proteins, which implement some role). In this paper, a **reference genome** is a good-quality PATRIC genome that serves as a template for extracting draft genomes from a sample; a **universal role** is a role that is expected to occur exactly once in any prokaryote.

The PATRIC service uses phenylalanine tRNA synthetase, alpha subunit (*pheS*) as its signature universal role. This protein is long enough (209–405 amino acid residues in Bacteria and 293–652 in Archaea) to provide sufficient resolution for distinguishing between organisms at the species level but sufficiently conserved to provide reasonable closest-neighbor estimates for undersampled clades. Both of the above considerations are relevant to the selection of reference genomes, which is essential to the success of the supervised binning method. In PATRIC, *pheS* is the most abundant single-copy role, so the set of potential reference genomes is slightly larger than for other potential universal roles. For a more thorough treatment of role abundances in PATRIC, refer to Table 1.

**Table 1:** Most common roles in PATRIC. The top 10 most commonly-occurring single-copy roles in PATRIC, along with their counts. *pheS* is consistently the most common role, whereas others shift around. *pheS* was chosen because of its abundance in PATRIC and good performance as a seed protein in the binning service.

Role	Count
Phenylalanyl-tRNA synthetase alpha chain (EC 6.1.1.20)	289,549
SSU ribosomal protein S15p (S13e)	288,826
LSU ribosomal protein L13p (L13Ae)	288,667
Ribonuclease HII (EC 3.1.26.4)	288,633
Phosphoglycerate kinase (EC 2.7.2.3)	287,460
Peptidyl-tRNA hydrolase (EC 3.1.1.29)	287,126
Histidyl-tRNA synthetase (EC 6.1.1.21)	286,464
SSU ribosomal protein S2p (SAe)	286,316
DNA-directed RNA polymerase alpha subunit (EC 2.7.7.6)	286,147
LSU ribosomal protein L6p (L9e)	285,951

We use *pheS* rather than more conventional 16S rRNA-based methods because the faster molecular clock and single copy number make inference easier. Several studies have shown that universal roles achieve equal or better performance than 16S rRNA at distinguishing between populations [8, 9]. In practice, *pheS* will likely underperform 16S rRNA for making inferences across large phylogenetic distances; since the binning service depends on identifying nearest neighbors only, this consideration should be largely immaterial.

It should be expected that, so long as the nearest reference for a given bin is sufficiently far away from the references for the other bins, even distant reference genomes will yield high quality bins. In any case, highly dissimilar reference genomes should happen when closer references are unavailable in PATRIC. This is empirically confirmed (See “Effect of *pheS* distance on bin quality” in Discussion section). Since the clinical significance of undersampled clades is largely unknown, the selection of pheS for supervised binning is consistent with the priorities of the PATRIC project and user base. Moreover, the expansion of the PATRIC database to include more genomes from undersampled clades should substantially reduce the incidence of such cases over time.

This implementation leverages the PATRIC database structure for draft genome reconstruction by accessing a large set of reference genomes and leveraging quality assessment tools like EvalG and EvalCon to verify draft genome quality. The binning service relies on a number of PATRIC-specific quality metrics (EvalG completeness, EvalG contamination, and EvalCon fine consistency) which we describe in a recent paper [6].

### Quality metrics

PATRIC uses a custom set of metrics for evaluating genome quality. In PATRIC, **completeness** is defined as the percentage of expected single-copy universal marker roles actually observed in a genome; this is equivalent to CheckM completeness. On the other hand, **contamination** is defined as the percentage of expected single-copy universal marker roles with observed counts greater than 1; this corresponds to the percentage of the sample roles occurring due to contamination. This is not the same as CheckM contamination [10].

Finally, **fine consistency** is a PATRIC-specific metric based on the EvalCon algortihm, which computes expected copy numbers for genes on the basis of a set of random forest predictors jackknife predictor trained on observed copy numbers of roughly 1,300 “reliably predictable” genes [6]. Reliably predictable genes are simply those for which such a predictor is capable of correctly predicting copy number more than 93% of the time, and the set of reliably predictable genes is maintained in PATRIC. Fine consistency is defined as the percentage of roles which are predictable via EvalCon whose observed counts match their expected counts exactly.

A high-quality genome is defined by the following quality cutoffs:

1. Contamination score less than or equal to 10%
2. Fine consistency score greater than or equal to 87%
3. Completeness score greater than or equal to 80%
4. Exactly one *pheS* gene of appropriate length (209–405 amino acid residues for bacteria, 293–652 for archaea).

### Dataset construction

We use all 193,980 high-quality genomes in PATRIC as reference genomes for the binning service. We note that all genomes from NCBI RefSeq are available as potential reference genomes, provided they meet the quality criteria described.

### Description of binning pipeline

**Fig 1:**
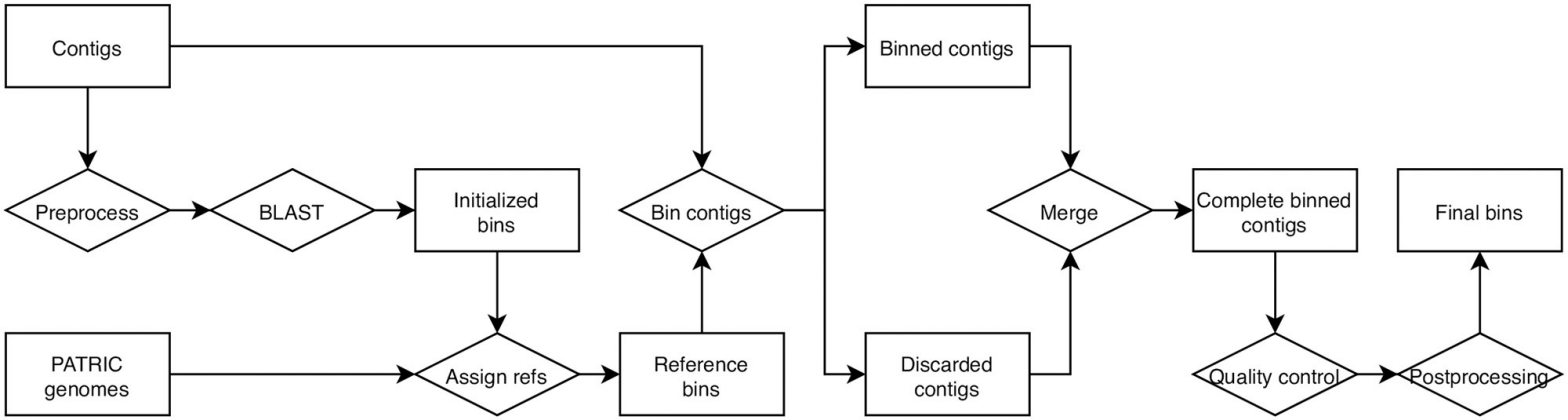
Flowchart of binning pipeline. This flowchart represents the process for creating bins from metagenomically-derived contigs. Bins are initialized on the basis of BLAST hits for a seed protein, then assigned references from PATRIC genomes. The remaining contigs are assigned to initialized bins on the basis of similarity to reference genomes, and then postprocessed in order to (1) relax stringent binning rules on the basis of similarity between an unallocated contig and a bin, and (2) to clean up bins with excess contamination.

Fig 1 lays out the steps of the binning pipeline which are described below.

#### Contig preprocessing

The contigs in the sample are first quality-controlled to eliminate long ambiguity runs and contigs under 400 base pairs in length.

#### Initialize bins

The binning service begins by identifying all occurrences of *pheS* in the sample by BLASTing the contigs in the sample against a small database of *pheS* sequences. Since this produces many low-quality hits by default, hits are discarded if they do not cover two-thirds of the *pheS* sequence, or if the target contig has less than 4-fold average coverage, or if the target contig is less than 400 base pairs in length. Once the low-quality hits have been discarded, each remaining bin is identified as a provisional bin.

A bin is identified by its *pheS* sequence and contains a set of contigs. At the end of the initialization step, there are *N* bins containing exactly one contig, namely the contig containing the *pheS* role. Each of these bins is expected to correspond to a single species in the sample, although bins may be merged in subsequent steps if they turn out to be closely related. The remaining contigs, which do not contain *pheS sequences, remain unbinned at this step.*

#### Assign reference genomes

The metagenome binning service uses a supervised binning method to sort contigs into draft genomes. It compares contigs to the reference set of genomes in order to place them into bins. By contrast, unsupervised methods will use intrinsic characteristics such as GC content to group contigs together. (At this point, only the contigs containing *pheS* instances will have been binned).

To assign reference genomes, the binning service compares the DNA sequences of the *pheS* instances in each bin to the reference set and chooses the closest match. Two bins are merged if their reference genomes belong to the same species. Users should be warned that, although subsequent steps are designed to clean up such artifacts, bin merging may produce contamination or chimerism in genome bins.

Next, sets of discriminating protein 12-mers are computed based on the reference genomes. A sequence of 12 amino acids in a given genome is said to be a discriminating protein 12-mer of that bin if it occurs in that bin’s reference genome(s) and does not occur in any of the other reference genomes. Discriminating protein 12-mers are specific to the given sample.

#### Bin contigs

A contig *C* is placed into the bin belonging to reference genome set *G* if *C* has at least 10 discriminating protein 12-mers in common with *G*, and no other reference genome has more discriminating protein 12-mers in common with *C*. Contigs failing to meet the minimum similarity of 10 discriminating protein 12-mers with any of the reference genomes do not get binned. This is done for each contig until all contigs are either binned or discarded.

Finally, the discarded contigs are checked for long DNA sequences (50 base pairs or more) exactly matching a single successfully binned contig from the previous step. If there is such a match between a discarded contig and a binned genome, the discarded contig is also placed into the corresponding bin. Sequences mapping to multiple references are not used in this step. Discarded contigs without such a match are never binned. In total, this will bin an additional 10-20% of the contigs.

#### Evaluate bin quality

Each bin created in the previous step represents a draft genome and can be annotated using the RASTtk pipeline. These annotations are used to compute three metrics of quality: completeness, contamination, and fine consistency. In addition to the quality metrics, each genome produced by the binning service includes a structured-language report on the absences or occurrences of problematic roles, i.e. roles which do not occur the expected number of times in a given genome. The quality metrics and problematic role report provide a check on the quality of the metagenome binning and allows users to review potential errors.

#### Postprocessing

For genomes showing high levels of contamination (over 10%), a final postprocessing step is applied wherein contigs that do not get annotated with any good roles during the annotation step are also discarded from the draft genome. During the annotation step, the EvalCon quality checker predicts *expected multiplicites* (i.e. the number of genes in a bin implementing a given role) using a leave-one-out random forest predictor for a set of reliably predictable roles. In this case, a contig having “no good roles” means that, for all of the *observed roles* found on that contig, the multiplicity predicted by EvalCon does not match the observed multiplicity of roles across the entire draft genome. When more occurrences of a role are observed in a given genome bin than predicted by EvalCon, the occurrences with the highest protein similarity to each occurence in the reference genomes are considered “good,” and the remaining occurrences are potential contamination. Contigs with no good roles tend to be short or misassembled, and discarding these contigs helps ensure that draft genomes correspond to general expectations about gene content in a given organism.

### Binning service outputs

The metagenome binning pipeline is integrated into the PATRIC website as a service. Users can upload assembled contigs or paired reads (in which case the reads are first assembled using metaSPAdes). The user must also specify an output folder and a name for this PATRIC job. No additional input is required. The binning service is designed to run with minimal user input and still provide state-of-the-art metagenome binning.

When completed, the metagenome binning job produces a job directory containing BinningReport.html, a structured-language HTML report on binning effectiveness, as well as FASTA files and annotation reports for each of the bins. Each bin spawns a separate annotation job, which can be viewed in the same way as a regular annotation job submitted through the PATRIC annotation service.

**Fig 2:**
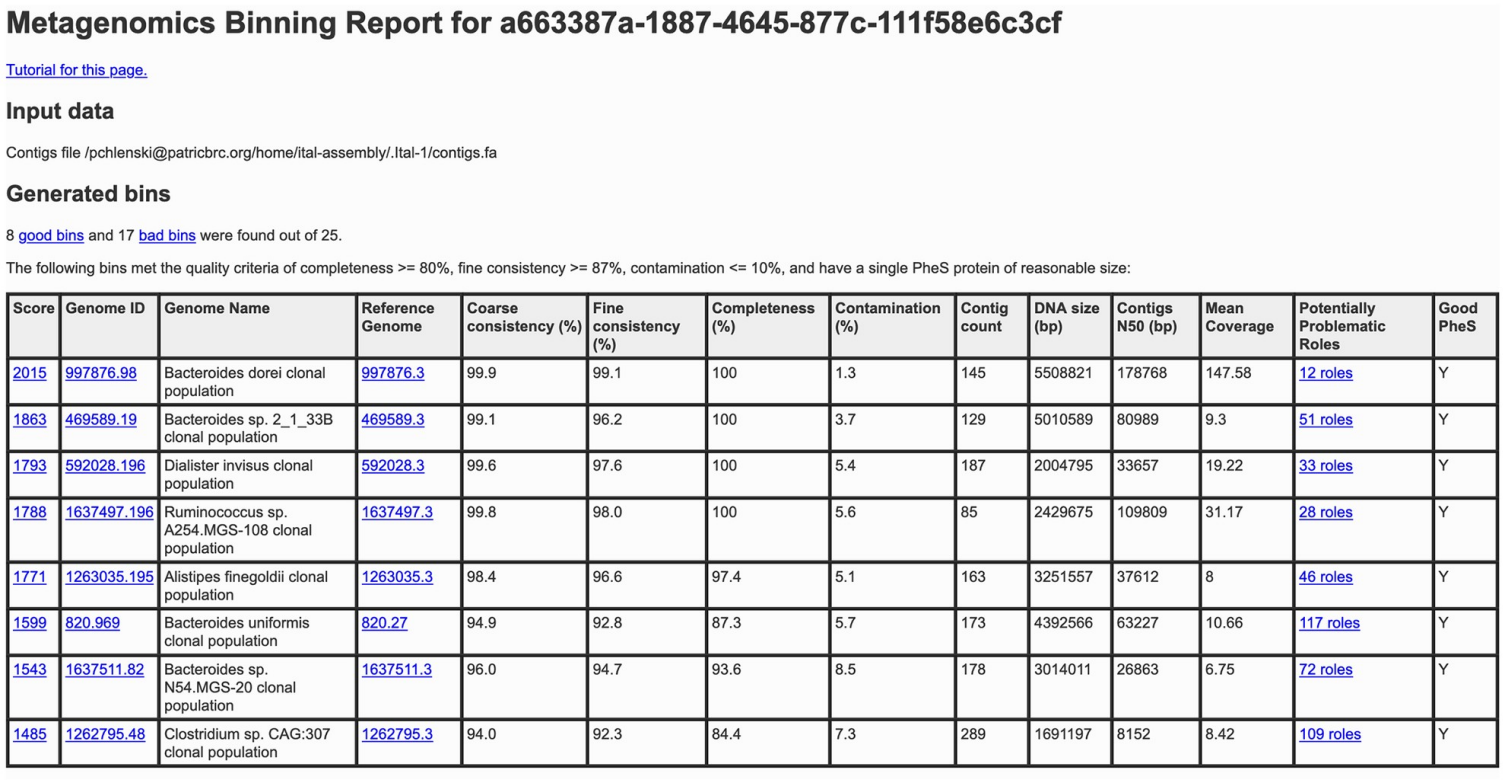
Metagenomic binning report for high-quality bins. The first part of the generated binning report summarizes the inputs, total number of bins, and quality breakdown of the bins. For high-quality bins, it reports the overall score, genome ID (with link to PATRIC genome page), organism name (ending in “clonal population”), a link to the reference genome used in the binning process, and a number of quality metrics including a link to the problematic roles report.

**Fig 3:**
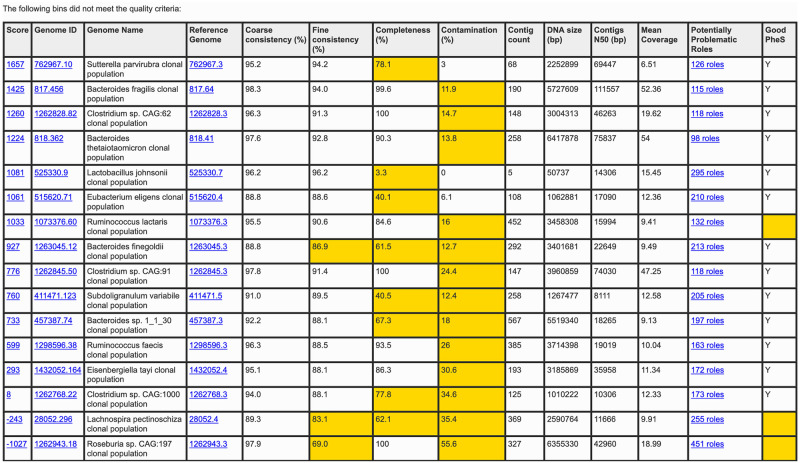
Metagenomic binning report for other bins. The second part of the generated binning report lists the low-quality bins found. The columns are the same as for the high-quality bins, but any metrics that fail to meet the criteria for a high-quality bin are highlighted in yellow. This gives researchers a starting point into identifying what may have gone wrong in the assembly, binning, and/or annotation process.

**Fig 4:**
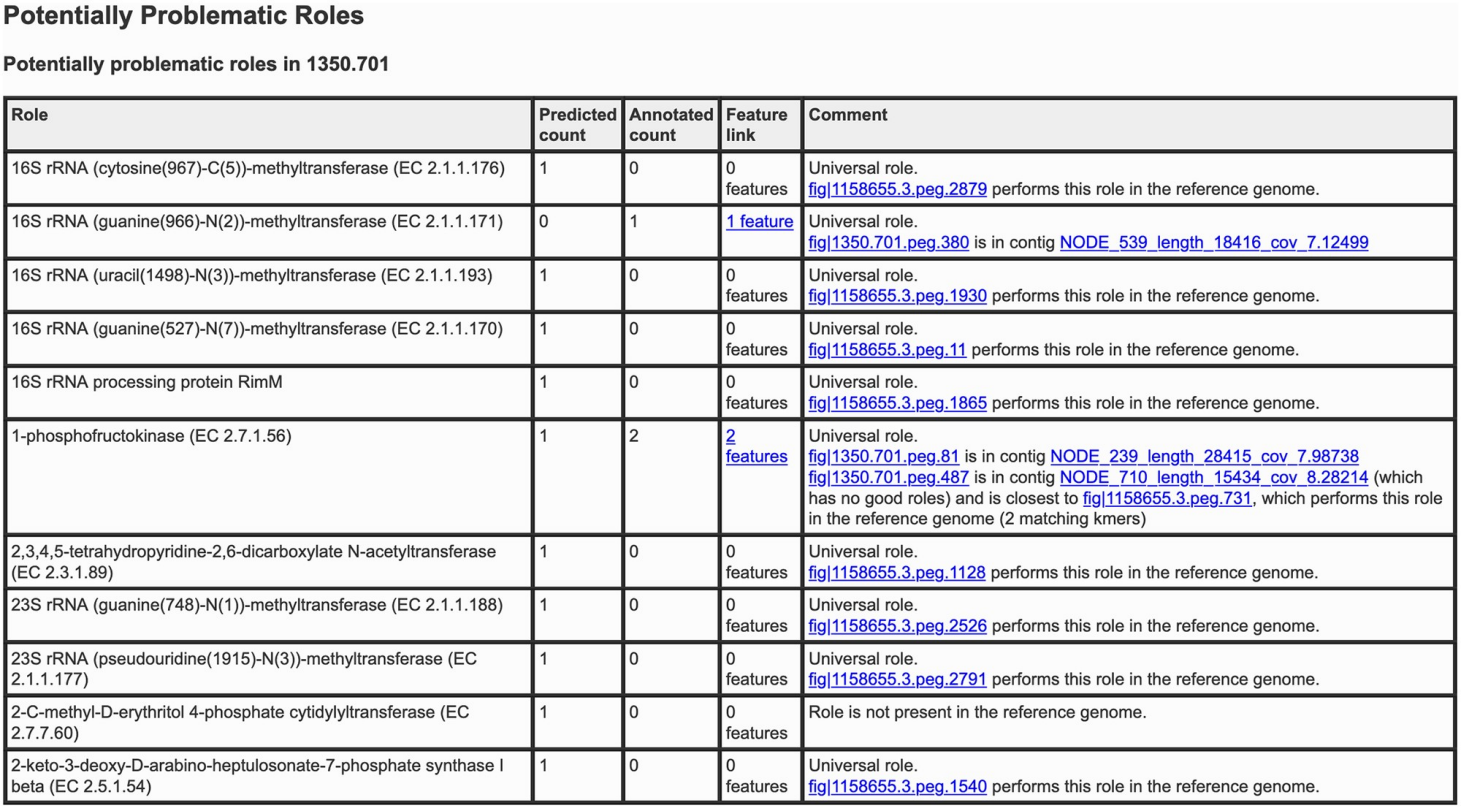
Sample problematatic role report. The problematic role report is generated per genome bin and reports on all genes whose observed multiplicity disagrees with PATRIC predictors. There are a number of reasons that a gene may appear in this report, including missing roles due to deletions, incompleteness, or frameshift mutations (observed multiplicity less than predicted); duplicate roles due to contamination or other binning issues (multiplicity more than predicted). The problematic roles report provides other information, such as whether a gene is known to be single-copy in general, and which genes have analogs in the reference genome. This information can help users discriminate between interesting variations from the reference genome and errors introduced in the binning, assembly, or sequencing stages.

The binning report contains information about the input file and a table of quality metrics (reference genome, coarse consistency, fine consistency, contamination, completeness, contig count, DNA size, contigs N50, mean coverage, high-quality *pheS*) and a link to the problematic role report. Any quality metrics falling below the high-quality genome cutoff are highlighted in yellow. Additional information on the use of this tool through the PATRIC website can be found in the metagenome binning service user guide and tutorial, both of which are linked on the upload page and accessible via the help page. Figs 2 and 3 show example binning reports; Fig 4 shows a problematic roles report.

The purpose of the binning report is to facilitate troubleshooting of the binning process and guide users’ interpretation of binning results and downstream analyses. Users are encouraged to consult the binning report and relevant problematic role reports prior to conducting downstream analyses or publishing binning results. In particular, the problematic roles report sheds light on how predicted gene copy numbers diverge from observed copy numbers and facilitate comparison with the relevant genes (when they exist) in the reference genome. Users who are interested in a more detailed description of the problematic roles report, how it is derived, and how it can be used to improve draft genomes further should consult [6].

## Results

### Effect of postprocessing

When run on a set of 639 metagenomic samples from SRA, the pipeline without the postprocessing step produces an average of 1.7 high-quality genome bins per metagenomic sample. After postprocessing, high-quality bin yield increased to 8.17 high-quality genome bins per sample.

### Effect of *pheS similarity* on bin quality

**Fig 5:**
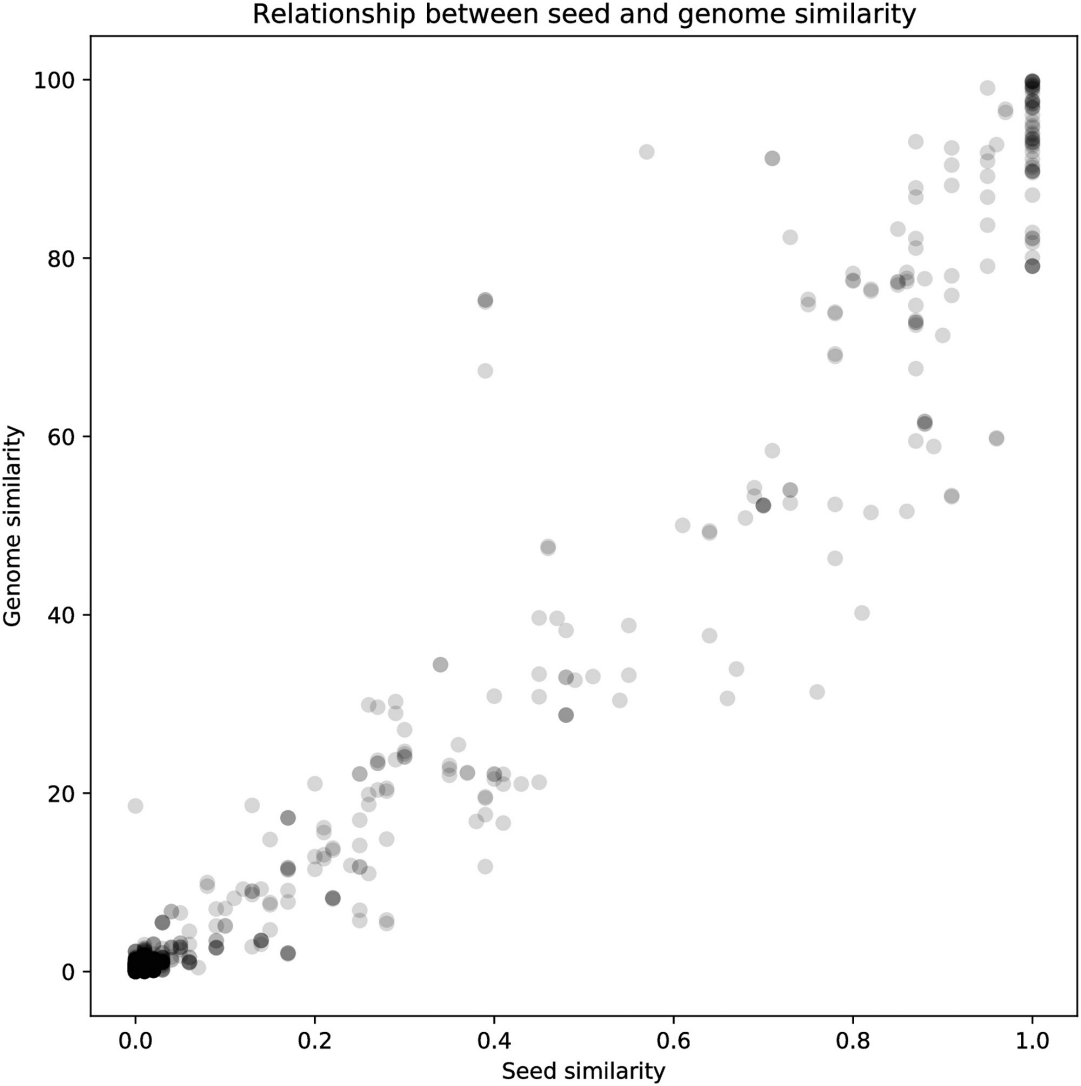
Relationship between distance to seed protein and genome similarity. This is a comparison used to determine the correspondence between *pheS* distance and mutations in the genome. The “similarity” number is the percent of known (i.e. annotated, non-hypothetical) roles that match. So, given one of the 11,533 roles in subsystems, if it occurs in both genomes, we compare the kmers in common to get a percent match. If it occurs in only one of the genomes, it is considered to have a similarity of 0%. This is all accumulated into a percentage protein similarity over the whole genome. If the similarity is listed as 0.50, note it means the match is one-half of one percent, not fifty percent. The pearson coefficient is 0.97, which means there is almost a one-to-one correspondence between role similarity in the genomes and *pheS similarity*.

To investigate the effect of a bin’s distance to its reference genome, we looked over 17,342 random pairs of genomes in PATRIC. The *pheS similarity*, which is defined as the Jaccard similarity between the sets of 8-mers represented in two *pheS* amino acid sequences, was compared to the overlap in roles (Fig 5). Similarity was defined as the percent of known (non-hypothetical) roles matching between two genomes. The Pearson correlation coefficient between *pheS* distance and genome similarity was 0.97, indicating a very strong correspondence between role overlap and *pheS similarity*.

Additionally, genome quality distribution by *pheS* distance reveals information about the limits of the binning service’s performance and the proportion of high-quality bins detected (Table 2). Of all 13,275 bins located by the binning service, 5,338 matched their reference *pheS* sequence exactly. Bins with a *pheS similarity* to their reference over 0.8 retrieved a high proportion of high-quality bins (54% of bins in this group were high-quality). No bin had a *pheS similarity* to its reference between 0.7 and 0.8. Bins with lower similarities performed substantially worse, but high-quality bins were retrieved at similarities as low as 0.3. In fact, the 0.3–0.4 similarity range shows an anomalously high rate of high-quality bins retrieved (21.5%).

**Table 2:** Relationship between seed similarity and genome similarity. The correspondence between *pheS similarity* on the number of high-quality and total bins found. In general, the further the reference is from the original genome, the more likely it is that the bin will not be high-quality. A *pheS similarity of 0.3* is sometimes smaller than a species and sometimes larger, but it is usually inside the same genus. In general, it should be expected that the binning service will do very well retrieving bins with *pheS similarity greater than 0.8*, and is able to retrieve high quality bins at distances as far as 0.3 in some cases. No high-quality bins were retreived with *pheS similarity below 0.3*.

Similarity	Good	Bad	Percent
1.0	3455	1883	64.72
0.9–1.0	1693	2001	45.83
0.8–0.9	649	1047	38.27
0.7–0.8	0	0	0.00
0.6–0.7	115	714	13.87
0.5–0.6	75	800	8.57
0.4–0.5	40	375	9.64
0.3–0.4	92	336	21.50

A similarity of 0.3 may or may not be in the same species as the reference, depending on clade of the organism, but it is almost always in the same genus. No high-quality bins with *pheS similarity below 0.3* were retrieved.

Thus, the binning service is capable of retrieving genomes with similarities of at least 0.3 from PATRIC reference genomes. This means that new strains and species can be retrieved with reference genomes at the genus level. Conversely, novel organisms lacking a reference genome at the genus level may not be binned, or may be binned into a draft genome of deficient quality.

### Performance benchmark

The PATRIC metagenome binning tool was developed for a different purpose than most metagenome binning tools. It is designed to recover as many high-quality draft genomes as possible from metagenomically-derived contigs for use in PATRIC rather than to correctly bin as many contigs as possible or retrieve the greatest number of bins from a sample. The PATRIC tool does not retrieve population abundances and may fail to extract novel or very low-coverage genomes, depending on the reference genomes available in PATRIC and the quality of the assembled contigs. Nevertheless, users may be interested in how PATRIC measures up against existing state-of-the-art metagenome binning tools, so we attempt to provide such a benchmark according to PATRIC quality criteria. Our intention is not to claim that our pipeline is better than other tools, but merely to show that it is useful for many common metagenomics use cases.

To establish a benchmark of the PATRIC binning performance compared to other tools, we ran a benchmark on 23 samples chosen at random from those used in a recent study by Pasolli *et al*. We selected these samples because the study compared a wide variety of human metagenomic samples, which is typical of the demands of PATRIC users working with metagenomic samples. We compared the following methods:

1. The PATRIC binning service, using only contigs from the Pasolli *et al* study
2. The Pasolli *et al* bins
3. A pipeline which involved downloading raw reads from SRA, aligning with Bowtie2, and binning with MetaBAT2 [11].
4. Binning with MetaBAT2 using only contigs from the Pasolli *et al* study

**Fig 6:**
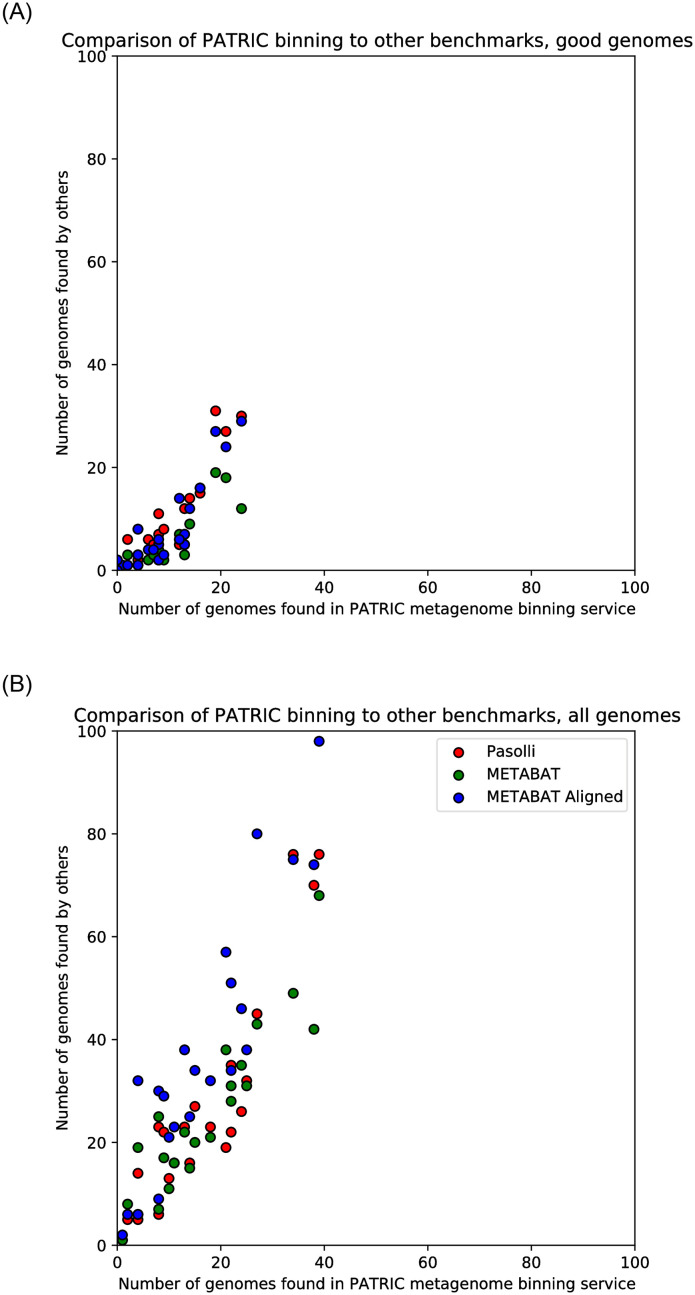
Comparison of PATRIC binning to other benchmarks. This plot indicates the number of total bins and high-quality bins (according to PATRIC criteria) generated by Pasolli *et al.* in their study, by our two benchmarks of MetaBAT2 (with and without alignments), and by the PATRIC metagenome binning service. Each point represents a sample; the left graph shows the number of high-quality bins found, while the right graph shows total bins found. Since Pasolli *et al.* is a good approximation of the state of the art in metagenome binning, this test suggests that the PATRIC binning method achieves performance close to the state of the art.

We chose Pasolli *et al*, which also uses MetaBAT2 for contig binning, as an example of MetaBAT2’s capabilities when running in a completely custom pipeline. The results are summarized in Table 3 and plotted in Fig 6.

**Table 3:** Benchmarks for various binning methods. 24 samples from the Pasolli *et al* study were run through the PATRIC binning tool, MetaBAT2 with depth files generated from the original FASTQ reads using Bowtie2, and MetaBAT2 on raw contigs. These bins were compared to the results generated in the Pasolli *et al* study via a custom pipeline which also used Bowtie2 and MetaBAT2. Each studied group splits found bins into “good” bins, i.e. those matching the PATRIC criteria for a high-quality bin, and “poor” quality bins which do not meet the criteria. In general, PATRIC found more high-quality bins than MetaBAT2 with or without alignment, although theoretically better MetaBAT2 performance is demonstrated via the Pasolli *et al* study. The PATRIC binning tool found substantially fewer poor-quality bins than any of the other binning methods.

	PATRIC		Pasolli et al		MetaBAT2 + Bowtie2		MetaBAT2	
Sample	Good	Poor	Good	Poor	Good	Poor	Good	Poor
ERR1136887	14	8	14	21	12	39	9	22
ERR1398081	13	11	7	19	7	39	5	30
ERR260232	4	4	8	15	8	22	3	22
ERR321564	21	18	27	49	24	74	18	50
ERR525795	8	10	7	16	5	27	2	19
ERR526044	6	5	6	10	4	19	2	14
ERR527062	8	7	11	16	6	28	4	16
ERR528311	7	7	5	11	4	21	3	12
ERR911992	16	11	15	30	16	64	16	27
ERR912091	19	15	31	45	27	48	19	30
ERR912124	24	14	30	40	29	45	12	30
SRR060006	8	2	5	8	2	19	2	9
SRR1950750	4	0	2	3	3	3	3	3
SRR1950766	1	0	0	1	1	1	0	1
SRR341647	0	4	2	12	2	30	2	17
SRR341697	2	7	6	16	1	28	3	14
SRR413750	12	9	5	14	14	43	7	31
SRR4305113	4	4	2	4	1	8	1	6
SRR4408221	0	2	1	4	0	6	0	8
SRR5091568	13	12	12	20	5	33	3	28
SRR5127609	12	10	5	17	6	28	6	22
SRR5279233	9	4	8	15	3	35	2	20
Totals	205	164	209	386	180	660	122	431

In total, Pasolli *et al.* found 387 genome bins, of which 210 are high-quality according to our definition. We found 370 bins, of which 206 were high-quality. Since the Pasolli *et al.* study discovered a wide range of previously undiscovered and unculturable species, their use of unsupervised binning gives them a major advantage in this comparison. We hope that integrating their data into the PATRIC database will result in better performance in undersampled clades.

MetaBAT2 with alignments discovered 840 bins, of which 180 were high-quality according to our definition. MetaBAT2 running on just the contigs discovered 533 bins, of which only 122 were high-quality. MetaBAT retrieves more bins, but the majority are of poor quality. High-quality genomes will have far more informative PATRIC annotations, so we believe retrieving more high-quality genomes is important for PATRIC users. Users who instead wish to retrieve as many bins as possible may prefer other binning methods, such as MetaBAT2.

MetaBAT2 is flexible and adaptive, eliminating the need for manual tuning that the original MetaBAT faced. However, the fact that our best MetaBAT2 pipeline still underperforms Pasolli *et al* benchmark casts doubt on the performance capability of an automated MetaBAT2 pipeline in the PATRIC environment for typical use cases. It is possible that the problem lies upstream in the pipeline (e.g. the alignments are suboptimal) and not with MetaBAT2. While both the PATRIC binning service and MetaBAT2 run on the order of minutes, generating the alignment files necessary for optimal MetaBAT2 performance can be computationally demanding for large read files. The tradeoff between performance and runtime is a consideration.

For users who are submitting raw reads to the binning service, we will consider the option of using Bowtie2 and MetaBAT2 for binning; for those who have only contigs to submit, we believe the PATRIC binning service provides superior results.

## Discussion

Because this service uses a supervised method, its effectiveness is highly dependent on the availability of high-quality reference genomes. Because the effectiveness of the binning service hinges on a binned genome being closer to its reference than to the references of all the other binned genomes, rather than simply on raw similarity, it is hard to characterize effect *a priori*. Nevertheless, the method is expected to underperform in undersampled clades, many of which may resist cultivation *in vitro*. Thus, reference genomes must be produced on the basis of metagenomic samples. On the one hand, increased attention to assembling and binning genomes from metagenomic samples, and in particular unsupervised binning, helps to characterize the diversity of metagenomic populations and fill in gaps in phylogenetic space. We expect the effectiveness of our supervised binning service to increase with the introduction of new references derived from unsupervised binning studies like Pasolli *et al*.

On the other hand, the lack of ground truth for metagenomically derived genomes is concerning. In particular, including metagenomically-derived genomes as references can proliferate errors in metagenomic assembly, binning, and annotation arbitrarily deep into undersampled clades. Contaminated assemblies and chimeric contigs in draft genomes can become established in the database as legitimate organisms, which would seriously affect the contig binning process. The metagenome binning service does not make users’ genomes public, and therefore they are never themselves used as reference genomes unless they are submitted to one of PATRIC’s public data sources. Currently, PATRIC’s quality metrics control the quality of reference genomes derived from other sources in order to avoid propagating assembly and binning errors into the binning pipeline, but further checks on metagenomic draft genomes may be needed.

In addition to the failsafes described above, PATRIC currently lacks a framework for dealing with metagenomically-derived bins or contigs as an aggregate, e.g. for the purpose of metatranscriptomics. The metagenome binning service currently creates a series of single-bin annotations and a genome group which supports some rudimentary multi-genome functionality, e.g. protein family heatmaps. Additionally, the current PATRIC metagenome binning service does not have tools for handling the presence of plasmids, phage and other viruses, or single-celled eukaryotes. These all represent potential areas for improvement of PATRIC’s support for metagenomics research.

Overall, the purpose of the PATRIC metagenomics service is to aid users in exploring metagenomics datasets and to provide high-quality draft genomes that can be used in downstream analyses. To make the most of the PATRIC binning service, users should make sure that their expecatations are aligned with this purpose and inspect the binning results (and in particular the problematic role reports) manually. Results from the binning service can be enhanced with downstream analysis of annotated draft genomes in PATRIC as well as by use of other metagenomics tools, e.g. METABAT2 for mining unbinned contigs for novel genomes.

## Conclusion

We have presented a service that takes paired-end reads or assembled contigs and produces a set of high-quality RASTtk-annotated contig bins. It additionally produces a report on the quality of all genome bins produced. This tool can be accessed via the PATRIC website through the Metagenomic Binning Service. We hope that the PATRIC binning service will serve many metagenomics needs of our user community and provide a valuable extension to the existing set of metagenomic contig binning tools.

## Supporting information

S1 File Binning summaries. This spreadsheet contains four pages, “Pasolli binning,” “Raw METABAT2 binning,” “Aligned METABAT2 binning,” and “PATRIC binning.” These are summaries of the bins found by Pasolli *et al*, alignment-based MetaBAT2, MetaBAT2 without alignment, and the PATRIC binning service, respectively. Each page reports on the fine consistency, completeness, and contamination of the bins, as well as whether they are marked “good” (i.e. high-quality) or “poor” by PATRIC.
(XLSX)

S2 File Contig allocation comparisons: Raw METABAT2 vs PATRIC. These are comparisons between PATRIC and the unaligned version of MetaBAT2. Each page in this spreadsheet, identified by an SRA accession number, summarized the allocations of contigs between METABAT2 and PATRIC.
(XLSX)

S3 File Contig allocation comparisons: Aligned METABAT2 vs PATRIC. These are comparisons between PATRIC and the aligned version of METABAT2. Each page in this spreadsheet, identified by an SRA accession number, summarized the allocations of contigs between METABAT2 and PATRIC.
(XLSX)

S1 Fig (TIF)

S2 Fig (TIF)

S3 Fig (TIF)
