# Supplementary figures and images for: Supervised extraction of near-complete genomes from metagenomic samples: A new service in PATRIC
Source: PLoS One. 2021 Apr 15;16(4):e0250092. doi: 10.1371/journal.pone.0250092 (PMC8049274; doi:10.1371/journal.pone.0250092)

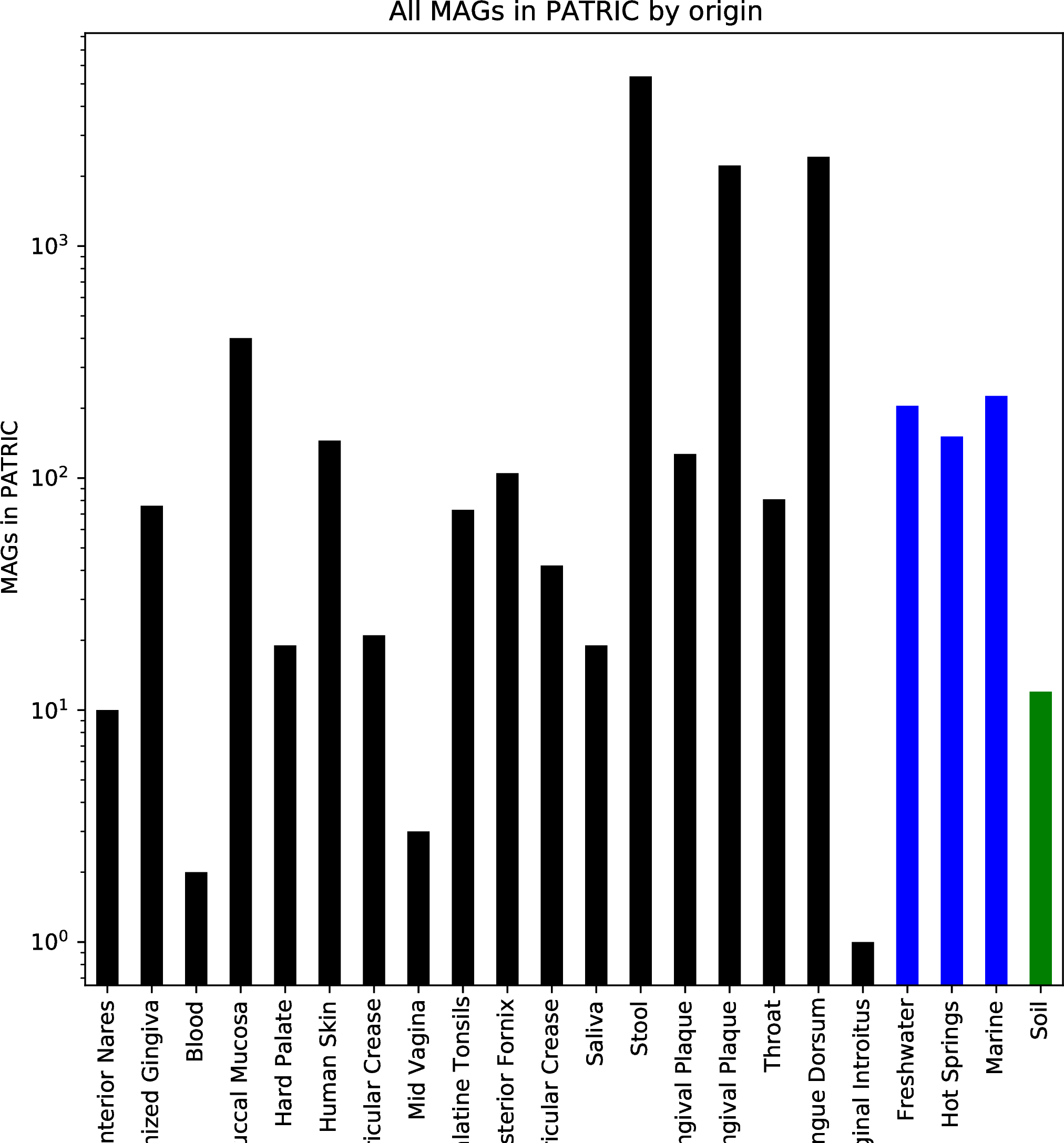

Supplement: S1 Fig — (TIF) [file pone.0250092.s004.tif]

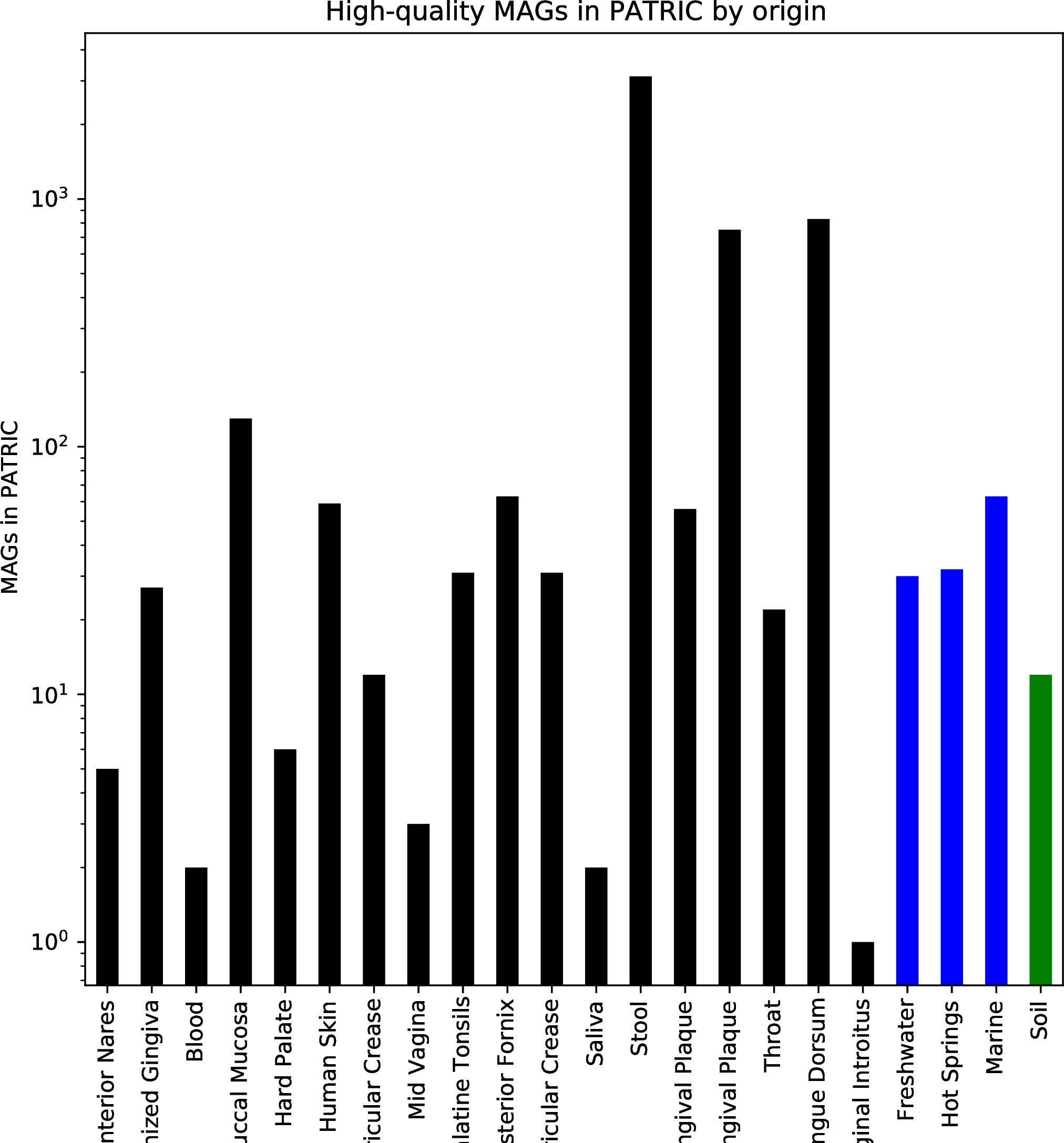

Supplement: S2 Fig — (TIF) [file pone.0250092.s005.tif]

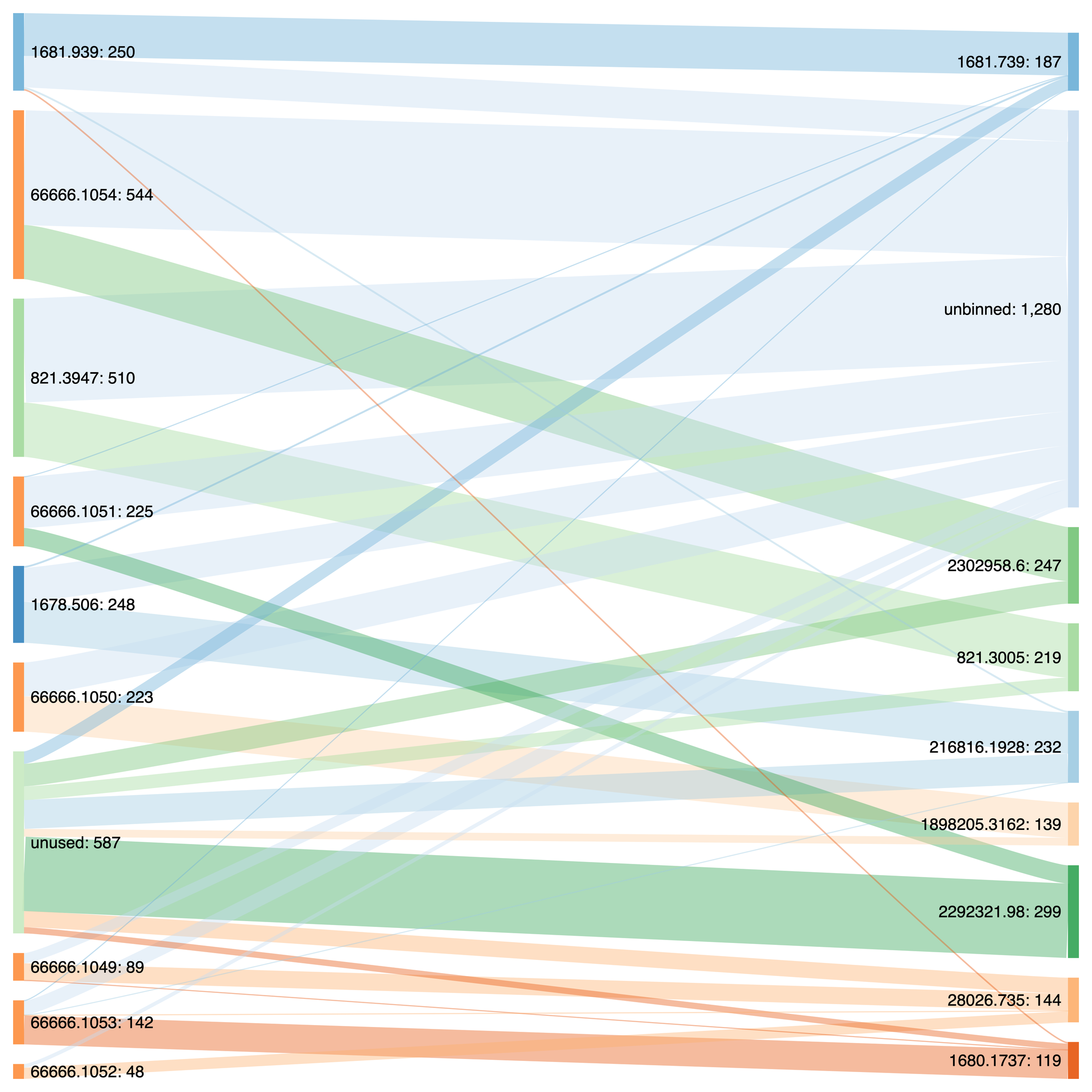

Supplement: S3 Fig — (TIF) [file pone.0250092.s006.tif]
